# Early‐life diet composition affects phenotypic variation of correlated animal personality traits

**DOI:** 10.1002/ece3.11567

**Published:** 2024-08-19

**Authors:** Eva Serrano Davies, Alba Miguel, Bernice Sepers, Kees van Oers

**Affiliations:** ^1^ Animal Ecology, Netherlands Institute of Ecology (NIOO‐KNAW) Wageningen The Netherlands; ^2^ Behavioural Ecology Group Wageningen University and Research Wageningen The Netherlands; ^3^ Department of Animal Behaviour Bielefeld University Bielefeld Germany

**Keywords:** behavioural plasticity, behavioural syndrome, great tit, multivariate analysis, parental care

## Abstract

Behavioural traits are under both genetic and environmental influence during early life stages. Early environmental conditions related to the amount and type of food have been found to alter behaviour in many organisms. However, how early life diet affects the variation in and the correlation between behavioural traits is largely unknown. Using a multivariate approach, we investigated how variation in parental prey selection is related to three repeatable nestling personality traits, and explored the within and between‐individual covariation between these behaviours in a wild passerine, the great tit (*Parus major*). Our results confirm that breath rate, docility and handling aggression (HA) in great tit nestlings are repeatable traits. Contrary to our expectation, the three nestling personality traits did not form a behavioural ‘syndrome’ on the phenotypic level in the study population, but we found two of three expected phenotypic correlations, mostly at the within‐individual level. Moreover, we found that breath rate significantly decreased with a higher number of spiders in the diet, and docility and handling aggression were significantly and inversely related to higher numbers of noctuids and tortricids in the diets of individuals within broods. Thus, our findings suggest that provisioning quantity and quality during the early life, affects variation in behavioural phenotypes, which occurs mainly at the within‐individual level.

## Graphical abstract

In nature, animal behaviour is both determined by the genes and the environment. The environmental conditions animals face in their early development, such as quality and quantity of food is known to alter behaviour. However, is diet composition related to the expression and relationship between behaviours? We here investigated how three nestling behaviours covaried and showed that different types of prey might determine the expression of particular behaviours in early life‐stages.

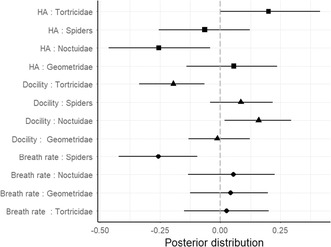

## 1 INTRODUCTION

A general question in evolutionary and behavioural ecology is how and by what mechanisms adaptive phenotypic plasticity in behavioural phenotypes arise. Consensus exists that although there is a significant genetic contribution to individual behavioural differences (Dochtermann et al., 2014; Laine & van Oers, 2017), also environmental factors influence behavioural phenotypes (e.g., Cole et al., 2021; Miranda et al., 2013), especially during the sensitive phase of early‐development (e.g., Carere et al., 2005). Early developmental effects on behaviour can be mediated by different environmental factors such as parasitism (Avilés et al., 2009), the social context (Naguib et al., 2011), or nutritional conditions (Stamps & Groothuis, 2010). Specifically, the latter, is known to play an important role in plastically shaping behaviour (Tremmel & Müller, 2013).

Early developmental nutritional variation may therefore affect behavioural responses of the offspring, extending to when they become adults (long‐lasting effects; Gabriel et al., 2005) and may have life‐long fitness consequences. This is especially evident when the early life environment is highly determined by the parents, such as in altricial species. For example, positive correlations between rearing environmental quality and behavioural phenotypes later in life have been found in traits such as exploratory behaviour (Serrano‐Davies et al., 2017), neophobia (Grunst et al., 2019; Miranda et al., 2013) and aggressiveness (Han & Dingemanse, 2017), but see (Nicolaus et al., 2019).

Given that parental prey selection and differences in diet plasticity (or environmental responsiveness) might modulate developmental trajectories via food provisioning (van Oers et al., 2015), individuals should feed on those resources most valuable for them or their offspring, as stated by the optimal foraging theory (Araújo et al., 2011). When providing care to the offspring, parental prey selection will not only cause variation in quantity of food but also in the quality, such as the variability of specific nutrients required during early development (Wu, 2014). On arthropod‐based diets, such as for several passerine bird species, the prey quality has often been attributed to protein content, which is a limiting nutrient under natural field conditions (Reynolds et al., 2003; Schoech & Bowman, 2003). For example, the relative contribution of bigger prey items to the diet, which entails higher protein content, has been demonstrated to be significantly related to nestling condition (García‐Navas & Sanz, 2010). But also certain amino‐acids, such as taurine, which is available in high concentrations in spiders (Ramsay & Houston, 2003) can affect development via a modulator role on neuronal activity and influence the nervous system development (Ripps & Shen, 2012). Both protein and amino‐acids content are known to have carry‐over effects on offspring behaviour (Arnold et al., 2007; Krause et al., 2009). In line with this, it has been shown that the supply of specific macronutrients can even have major pleiotropic effects on suites of behaviours (Han & Dingemanse, 2015).

Consistent among‐individual differences in behavioural traits (‘personality traits’, Réale et al., 2010) that are shown to covary across time or contexts, may form so‐called behavioural syndromes (Bell, 2007; Sih et al., 2004). In order to understand the evolutionary potential of the among‐individual differences in personality traits and the structure of a particular behavioural syndrome, it is necessary to partition the phenotypic correlations into their variance components and assess their relative contribution (Cleasby et al., 2015; Dingemanse et al., 2010). Variance decomposition of phenotypic correlations results in among‐individual correlations, which implies that consistent differences in trait mean exist, and within‐individual (residual) correlations, which implies environmentally induced phenotypic change (plasticity) (Brommer, 2013; Dingemanse, Kazem, et al., 2010).

Further, as theory predicts that correlated traits might coevolve in response to selection (Arnold, 1992), this suggests that behavioural syndromes could differ depending on the environmental pressures taking place for a particular population. Despite several studies have addressed the question of how parental early nutrition during the nestling phase affects the development of animal personality traits (Arnold et al., 2007; Sinn & Moltschaniwskyj, 2005; van Oers et al., 2015), whether differences in early life diet composition affects correlated behaviours in comparable ways remain unknown.

Therefore, in this study we aim to assess how variation in diet provisioning is associated to phenotypic differences in behavioural phenotypes, as well as to explore the within and among‐individual covariances between these behavioural traits in a wild passerine, the great tit (*Parus major*). We focused on multiple measures of three nestling personality traits that have been demonstrated to be heritable and to form a syndrome in blue tit fledglings, showing evidence of individual‐genetic differences in the sensitivity to stress (Brommer & Kluen, 2012): breath rate, docility and HA. We evaluated the existence of this syndrome in our study population by partitioning the phenotypic covariance matrix into individual and residual covariances, in order to explore whether the phenotypic correlations imply truly intrinsic covariances (among‐individual levels) or mostly capture residual (within‐individual) effects.

Besides, research focussing on nutritional stress during the nestling period in song birds has revealed effects on exploration, neophobia, risk‐taking and foraging in the long term when individuals were provided with low‐quality food compared to those that received higher‐quality food. For example, Krause et al. (2009) showed that zebra finches (*Taeniopygia guttata*) raised under low quality nutritional conditions were faster in exploration and foraging when tested as adults; and in the same lines, Arnold et al. (2007) found lower neophobia, and increased risk taking behaviour when comparing supplemented blue tit (*Cyanistes caeruleus*) nestlings to control broods. Variation in quality and quantity of food provided by parents may originate from the fact that parents consistently differ in their prey preference (Costantini et al., 2005) due to intrinsic traits such as morphology or behaviour (fundamental specialisation). Alternatively, they might vary in actual resource use as a result from extrinsic mechanisms (resource patchiness, social interactions; Bolnick et al., 2003). Previous studies on Parids have shown both a species‐specific preference of caterpillar type as well as a behavioural flexibility in provisioning strategies to cope with changing scenarios. For instance, great tits showed an overwhelming preference for noctuids, large caterpillars but difficult to find as they feed externally on the upper or lower leaf surface, which makes them less conspicuous from the distance in comparison to tortricids, which are smaller caterpillars but easier to find as they feed within a shelter constructed by rolling one or more leaves in a tubular fashion (leaf‐ rollers), which were the most common prey for blue tits (García‐Navas et al., 2013). Also, as a result of a short‐term brood manipulation experiment in blue tits, parents from the enlarged broods (high level of feeding responsibility) opted to concentrate on more readily available food items (tortricids), while the response of those from reduced broods (a low level of feeding responsibility) was to decrease this prey type in the diet, so that the percentage of other preys (noctuids) in the diet increased (García‐Navas & Sanz, 2010). Based on this, in the same line of reasoning, we expected that diet composition will be related to personality traits on the phenotypic level. Thus, nestlings that were provisioned with a higher quality diet regime, including a higher proportion of the preferred prey types (spiders and noctuids) will show less stress (lower breath rate and lower docility) and will be more aggressive than those receiving lower quality food, which would include a higher proportion of the non‐preferred prey types (tortricids and geometrids).

## 2 MATERIALS AND METHODS

### 2.1 Study area and monitoring

Data were collected in spring 2019 from a long‐term study nest box population of great tits in the Westerheide estate (58500 E, 528000 N), Groot Warnsborn near Arnhem, The Netherlands. Westerheide is a mixed wood covering 100 ha with approximately 230 nest‐boxes. Nest‐boxes are routinely checked throughout the breeding season to determine hatching date of the nestlings (measured as the day the first egg in a brood hatched, in days from 1st of April). We captured both parents when their nestlings were 9–10 days old. They were ringed with a unique metal ring, measured (i.e., body weight to the nearest 0.1 g, tarsus to the nearest 0.1 mm) and released immediately afterwards. The nestlings were ringed and their biometry measured 14 days after hatching. We observed no hatching asynchrony in our broods.

All experimental protocols were approved by the Central Authority for Scientific Procedures on Animals (CCD Project AVD‐801002017831 to KVO) and the Animal Welfare Body (NIOO‐IvD). Captures were performed under personal ringing permits delivered by the Vogeltrekstation (Dutch Centre for Avian Migration and Demography).

### 2.2 Parental provisioning behaviour and diet characterisation

When nestlings were 11 days old, an infrared video camera was attached inside the nest‐box and focused to its entrance in order to record adult provisioning behaviour and to identify prey items. Broods were recorded for at least 150 min between 08:00 and 12:00 h. We discarded the first 60 and the last 30 min of each recording to standardise for possible disturbances caused by the camera installation and removal. Therefore, we analysed the remaining 60 min, which has been demonstrated to be sufficient to describe parental provisioning behaviour (Pagani‐Núñez & Senar, 2013).

Recordings were analysed using Open shot Video Editor V2.4.4 software, which allows slow‐motion and frame‐by‐frame playback. From scoring the videos we determined the number and type of prey brought to nestlings per individual parent per nest for 1 h. Following Serrano‐Davies and Sanz (2017) we distinguished eight main prey items fed to nestlings, included in two groups: lepidopteran larvae, which include noctuids (cutworms, 34% of the classified items), tortricids (leaf‐rollers, 16%), geometrids (inchworms, 25%), and undetermined larvae (9%), and non‐lepidopteran larvae which include spiders (2%), diptera (3%), undetermined (9%) and ‘others’ (2%), which groups infrequent prey and/or prey not included in the other categories (e.g. plant material, Coleoptera, Hemiptera, Hymenoptera, Orthoptera and adult Lepidoptera). We included noctuids, tortricids and geometrids in our subsequent analyses, as they represent 75% of the total diet and are the three most common lepidopteran families in the great tits diet (Royama, 1970), as well as spiders, which have been described to be an essential part of the diet in Parids (Blondel et al., 1991; Cowie & Hinsley, 1988; García‐Navas et al., 2012) and their behavioural development (Arnold et al., 2007).

### 2.3 Behavioural assays

We quantified three offspring behaviours during handling, when nestlings in each nest were 9 and 14‐days‐old following the methodology described by (Brommer & Kluen, 2012). Therefore, each nestling behaviour was measured twice. All nestlings were taken from the nest box and put together in a cotton bird bag. Nestlings were picked from the bag one‐by‐one and held in the hand loosely on their back, fixing them by keeping the head between thumb and index finger (Fucikova et al., 2009). We quantified breath rate (BR) by counting how many times an individual breathes per minute, using a stopwatch. Breath rate is a non‐invasive measure of the primary stress response, whereby a more rapid breath rate is indicative of a higher handling stress response (Carere & Van Oers, 2004; Fucikova et al., 2009; Krams et al., 2014). The handling period was divided into four 15‐s bouts, therefore obtaining four data points per minute. To obtain individual estimates for the handling stress response on days 9 and 14, we used a similar method as described in Fucikova et al. (2009) and van Oers et al. (2015). We ran two separate linear mixed models (*lmer* in r package *lme4* v1.1.30.), one for each of the measurement days (days 9 and 14), with the number of breaths per 15‐s bout as dependent variable. We included individual as a random effect, and weight, tarsus length, time, temperature, temperature squared and date of testing as covariates. We included the interaction between individual and bout as a fixed effect to produce an estimate for every individual, which refers to the individual deviation from an average slope in breath rate for further analysis. During the same handling events, we quantified docility, measured as the number of struggles during 1 min. Docility is defined here as an individual's reaction to being trapped and handled. It is a commonly used metric of personality and is often used as a measure of risk‐taking behaviour (Careau et al., 2010; Réale et al., 2007). We calculated docility as 10—the number of struggles per minute, such that a higher docility score indicated an individual which was more docile (i.e., struggled less).

We then measured handling aggression of the nestlings while fitting them with a metal ring and taking morphological measurements: tarsus length using a sliding calliper (±1 mm; only measured on day 14) and weight with a digital pocket scale (±0.1 gr). handling aggression reflects aggressive and distress behaviour in response to manipulation by humans (Senar et al., 2017). To quantify handling aggression, we used a score (ranging from 0 to 5) reflecting whether the individual is passive or aggressive when held by the observer. Thus, a bird that was completely passive during all measurements would score ‘0’, while a bird that struggled or pecked during the whole time of measurements would score ‘5’ (see Brommer & Kluen, 2012). We could not measure handling aggression of 13 individuals out of 259 on day 9 as they were not responsive to the test or the measure was not completely clear to us, therefore we decided to not give a score to those individuals. After the three behaviours were scored and measurements were taken, we put the individuals already tested into a cotton bag and, once all of them were tested, we put them back in the nest box.

### 2.4 Statistical analyses

In order to quantify the repeatability of the three behavioural traits, the between trait covariations and phenotypic divergence related to diet composition in great tits we fitted a multivariate mixed‐effects model using MCMCglmm (version 2.34; Hadfield, 2010). The model included the breath rate estimates, docility and handling aggression as the three response variables, fitting a Gaussian distribution for the first two and an ordinal distribution for HA. We included random intercepts for individual and genetic nest box identity. Moreover, to evaluate the relationship between diet composition and the phenotypic variance of each of the three behaviours, we included the number of items of the three most abundant caterpillar types (Noctuidae, Geometridae and Tortricidae) and spiders per nest in the model as fixed effects. Hence, the effects of the diet on behaviours were inferred on the between‐brood level and not at the between‐individual level. We used a parameter expanded and weakly informative prior for our model (*V* = 3, nu = 1.002; Gelman, 2006; Houslay & Wilson, 2017) and ran 750,000 iterations with a burn‐in of 50,000 and a thinning interval of 175 (effective sample size = 4000). All trace plots were visually inspected to ensure the mixing of chains and the absence of autocorrelation between posterior samples. The effect of a given predictor was considered significant if zero was not included in the 95% credibility interval (CI). The analysis and associated figures for this study were generated using R v. 4.2.2 (R Core Team, 2022) and R studio v. 2023.09.0 + 463.

This multivariate model allowed us to estimate the repeatability and variance of each trait, the phenotypic covariation between the three traits and decomposes these into their among‐ and within‐individual components (Dingemanse & Dochtermann, 2013). We calculated the ‘adjusted repeatability’ (i.e., the repeatability conditional on the fixed effects) for each trait by dividing their among‐individual variance estimates ($V_{I}=V_{A}$[additive genetic variance] $+V_{\mathrm{PE}}$ (permanent environment effect)) by the sum of their among‐individual and residual variances ($V_{P}=V_{A}+V_{\mathrm{PE}}$ $+V_{R}$ [within‐individual variance]). We retained information on individuals with only one measure of the behavioural traits performance in our repeatability calculations (Nakagawa & Schielzeth, 2010). The number of repeated measures per individuals for each behavioural trait was two. As such, repeatability was estimated as follows:

$$R=\frac{V_{I}}{V_{P}}=\frac{V_{A}+V_{\mathrm{PE}}}{V_{A}+V_{\mathrm{PE}}+V_{R}}$$

We calculated the phenotypic correlations between pairs of traits (breath rate‐docility, breath rate‐ handling aggression and docility‐HA) by dividing the covariance between each two traits by the product of the square root of their variances, i.e.:

$$\begin{array}{c} \text{Phenotypic correlation}=\\ \;\frac{{\mathrm{COV}}_{\text{trait}1,\text{trait}2}}{\sqrt{\left(V_{A,\text{trait}1}+V_{\mathrm{PE},\text{trait}1}+V_{R,\text{trait}1}\right)\times \left(\;V_{A,\text{trait}2}+V_{\mathrm{PE},\text{trait}2}+V_{R,\text{trait}2}\right)}} \end{array}$$

Among and within‐individual covariances were extracted from the model outcome following (Houslay & Wilson, 2017).

Through this model we also obtained the results regarding the diet composition fixed effects on the behavioural phenotypes. These are expressed as posterior mean of the posterior distribution, 95% credible interval and a pMCMC value, which is twice the posterior probability that the estimate is negative or positive (Hadfield et al., 2013), and provides a degree of significance for the parameter being away from zero.

## 3 RESULTS

### 3.1 Repeatability of nestling behavioural traits

A total of 259 nestlings, originating from 40 broods, were tested for the three behaviours. Handling aggression varied from 0 to 5 (mean ± SD: 1.693 ± 1.226), breath rate estimates from −3.803 to 7.391 (mean ± SD: 0.495 ± 1.614) and docility from 0 to 10 struggles/min (mean ± SD: 8.164 ± 2.162).

The repeatability of breath rate was 0.257 (CI = 0.132–0.391) while for docility it was 0.103 (CI = 0.016–0.204). The statistical support for handling aggression to be repeatable was weak, as the 95% lower CI limit was almost zero (*r* = 0.144, CI = 0.0002–0.291; Table 1). This finding implies that either handling aggression was not repeatable or that the statistical power to estimate its repeatability was insufficient. Within‐individual variance, which might include sampling error, exceeded among individual variance in all cases (Table 1).

**TABLE 1:** Number of samples, estimates of variance components and repeatability for breath rate, docility and handling aggression behaviours in the 9‐and 14‐day‐old great tits studied population, obtained from the multivariate model.

	Breath rate	Docility	Handling aggression
No. of measures	518	518	505
No. of individuals with replicated measures	259	259	246

	Estimate	95% CI	Estimate	95% CI	Estimate	95% CI
Variance components						
Among–individual (*V*_I_)	**0.257**	**0.119–0.404**	**0.104**	**0.009–0.203**	0.164	0–0.351
Within–individual (*V*_R_)	**0.722**	**0.604–0.839**	**0.891**	**0.770–1.031**	**1**	**1–1**
Repeatability	**0.260**	**0.132–0.391**	**0.103**	**0.016–0.204**	**0.144**	**0.0002–0.291**

*Note*: The table gives the mean posterior distribution and its 95% credible interval (CI). 95% CIs considered significant (that do not cross zero) are in bold.

### 3.2 Correlation between traits

From the (co)variance matrices obtained from the multivariate model, correlations between all traits were calculated on the phenotypic, the among‐ and the within‐individual levels (Table 2). There was a moderately positive and significant phenotypic correlation between breath rate and docility (Table 2), indicating that fledglings with a higher breath rate also were more docile. The decomposition of this phenotypic correlation revealed that this was not driven by an among‐individual correlation, but by a within‐individual correlation instead (Table 2). This means that an individual's change in breath rate across samplings is positively correlated with its change in docility across that same sampling event. On the contrary, there was an average negative and significant phenotypic correlation between docility and handling aggression (Table 2), indicating 0that more docile birds were also less aggressive. Also, this relationship was driven by within‐individual covariance (Table 2). We did not find evidence for a phenotypic correlation between breath rate and handling aggression (Table 2). The among‐individual and within‐individual levels showed the same sign and similar CI than the phenotypic level for these pair of traits.

**TABLE 2:** Phenotypic, among‐ and within‐individual correlations between breath rate, docility and handling aggression scores.

Correlation	Phenotypic		Among individual		Within individual	
	Mean	CI	Mean	CI	Mean	CI
Breath rate—docility	**0.186**	**0.093, 0.268**	−0.013	−0.085, 0.062	**0.195**	**0.107, 0.284**
Breath rate—HA	−0.089	−0.215, 0.039	−0.007	−0.108, 0.099	−0.085	−0.218, 0.042
Docility—HA	**−0.317**	**−0.438, −0.204**	−0.042	−0.124, 0.030	**−0.300**	**−0.427, −0.167**

*Note*: Posterior means and 95% credible interval (CI) are shown. Bold indicates estimates whose 95% CI do not encompass zero.

Abbreviation: HA, handling aggression.

### 3.3 Phenotypic divergence in behaviour and diet composition

**FIGURE 1:**
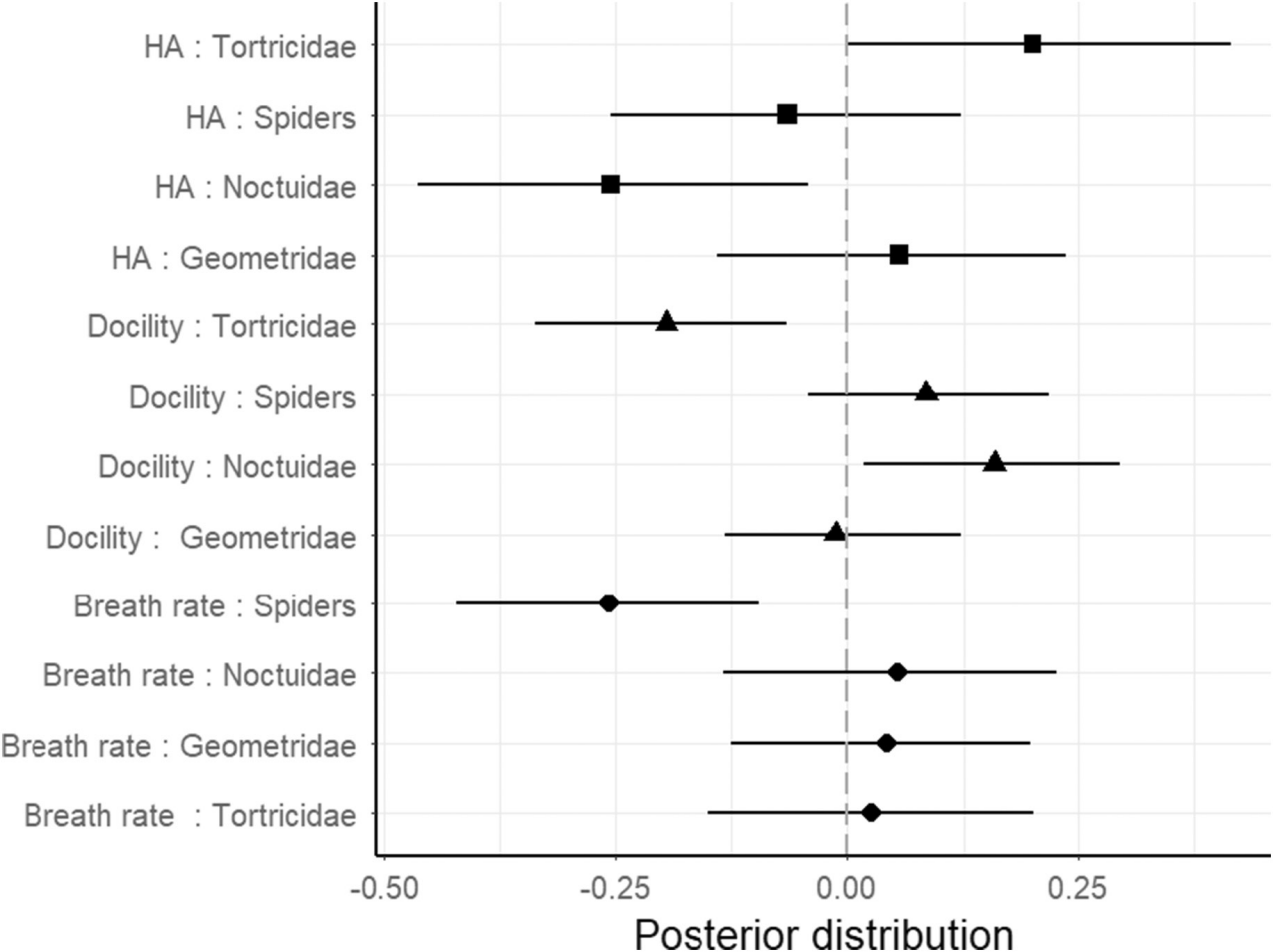
Effect of prey types (independent variables) on the three focal behavioural traits of great tit fledglings. Handling aggression (HA) states for HA. The posterior distribution of an independent variable with a negligible effect on a trait is expected to be centred on zero (dashed line); conversely, the distribution of an influential variable is expected to be shifted from 0. Different symbols to each response variable. Black squares (HA), triangles (docility) and circles (breath rate) indicate the mean of the posterior distribution, and horizontal lines indicate the 95% CIs.

The prey types included in the models, represented: Noctuidae 34% (min–max no. of items: 2–39), Geometridae 25% (0–31) and Tortricidae 16% (0–23) and spiders 2% (0–3) of the total prey items provisioned to the offspring. Breath rate associated significantly with the number of spiders in the diet (posterior mean = −0.257, CI = −0.422, −0.094, *p* = .003), with lower breath rates when the number of provided spiders increased. None of the other predictors in the model were related to breath rate (Figure 1; Table S1). On the other hand, variation in docility was significantly explained by the type of caterpillar that was delivered to the nest. Nestlings were more docile when there were more noctuids in the diet (posterior mean = 0.158, CI = 0.017, 0.295, *p* = .025; Figure 1), while on the contrary, they were less docile when tortricids number was higher (posterior mean = −0.196, CI = −0.337, −0.065, *p* = .007; Figure 1). None of the other predictors in the model were related to docility (Figure 1; Table S1). Lastly, handling aggression significantly associated with the same caterpillar types as docility, but in the opposite direction. Thus, handling aggression response of the nestlings was higher when parents provisioned them with a higher abundance of tortricids (posterior mean = 0.200, CI = −0.001, 0.413, *p* = .047; Figure 1), and it was lower with a higher abundance of noctuids (posterior mean = −0.254, CI = −0.463, −0.042, *p* = .015).

## 4 DISCUSSION

To investigate how differences in early life behavioural phenotypes are correlated between them and how they associate to parental prey selection, we here estimated the within and between‐individual variation of three stress‐related behavioural traits in great tit nestlings, and explored whether variation in parental prey selection, via diet composition, was associated with behavioural traits. The three studied traits were significantly repeatable, despite the statistical support for handling aggression to be repeatable was weak. However, we only found two low significant correlations between them, suggesting they did not form an integrated behavioural ‘syndrome’. Overall, our results showed that breath rate and docility formed a syndrome, as well as docility and HA, and that diet was related to the development of behavioural traits, so that the number of spiders in the diet, as a source of taurine, as well as the caterpillar type delivered modulated offspring behaviour.

In accordance with earlier studies on stress‐related behavioural traits (Caizergues et al., 2022; Fucikova et al., 2009), we found that breath rate, docility and handling aggression showed significant repeatability estimates in the study population, which suggests that these traits are consistent over time. Despite, in general, individual behaviours are often relatively inflexible (average repeatability of 37%, Bell et al., 2009), our repeatability estimates for breath rate are a bit lower than expected but lay within similar values than in previous results in the same population (0.33, Fucikova et al., 2009), other great tit populations (0.44, Caizergues et al., 2022) and for similar species (0.18 in blue tits, Kluen et al., 2014); and the same applies for docility (0.17 in juvenile yellow‐bellied marmots, *Marmota flaviventris*, Petelle et al., 2013; 0.40 mean docility in juvenile eastern chipmunks, *Tamias striatus*, St‐Hilaire et al., 2017). However, in the case of HA, repeatability was lower than previously reported in juvenile great tits (0.38; Caizergues et al., 2022) and blue tits (0.40; Kluen et al., 2014), and the 95% lower CI limit was almost zero, therefore close to non‐significance. We argue that this suggests that these traits might be highly influenced by the developmental trajectories, and point to differences in performance due to big changes in the behavioural development of juveniles between measures (9 days old vs. 14 days old). It is also important to mention that in our case (*N* = 259, two samplings per behavioural trait) the power to significantly identify nonzero values of repeatability is close to 1 for breath rate, while in the case of docility and handling aggression was around 0.30 (Dingemanse & Dochtermann, 2013). Future studies should include higher number of samples per individual in order to increase the power of the repeatability estimations.

Our design allowed us to evaluate the existence of a behavioural syndrome by partitioning the phenotypic covariance matrix into individual and residual covariances, which is crucial to ascertain whether a phenotypic correlation captures truly intrinsic covariances (between‐individual levels) or captures residual (within‐individual) effects. As hypothesised, breath rate and docility showed a moderately positive significant phenotypic correlation and there was also a negative significant phenotypic correlation between docility and HA. However, the phenotypic correlation between breath rate and handling aggression was quite small and non‐significant. In addition, after the decomposition of both significant, as well as the non‐significant, phenotypic correlations, all among‐individual phenotypic correlations between pairs of traits had credibility intervals overlapping 0 (Table 2), and they were driven by within‐individual correlations. This suggests that contrary to our expectations, the three focal traits did not seem to covary in an integrated behavioural syndrome. This is in contrast to Brommer and Kluen study (Brommer & Kluen, 2012) in blue tits, in which we based our hypothesis. Using a brood cross‐foster experiment for 3 years in blue tits the authors found both phenotypic and genetic correlations between them all, as well as being heritable. In particular, a significantly negative phenotypic correlation of −0.28 and a significantly negative genetic correlation of −0.50 between breath rate and handling aggression were reported. In the same line, other phenotypic correlations between breath rate and other personality traits such as boldness (Carere & van Oers, 2004) and exploratory behaviour (Fucikova et al., 2009), have been recorded previously. Also, physiological responses to stress, such as an increase in breath rate, through activated hypothalamo‐pituitary–adrenal (HPA) axis and elevated glucocorticoid (e.g. corticosterone) levels, have been shown to vary among individual animals typed as ‘proactive’ or ‘reactive’ and affect variation in behavioural trait responses among individuals (Koolhaas et al., 1999). On the other hand, other studies found no relation between breath rate and several personality traits in captive zebra finches (*Taeniopygia guttata*, David et al., 2012), or even no relation with handling aggression in great tits (Caizergues et al., 2022). Hence, in this study, great tit fledglings with a higher breath rate (more stressed by the handling procedure) were more docile, and more docile individuals were less aggressive, but selection acting on one of the focal traits is unlikely to induce indirect selection on the other traits. Instead, the phenotypic correlations were the result of a strong within‐individual correlation, which implies that underlying environmental factor(s) co‐modulate behavioural changes within the same individual over time.

Despite the considerable amount of research focused on the key role of diet composition during early development on shaping behavioural traits (e.g. Arnold et al., 2007; Brust et al., 2014; Tremmel & Müller, 2013; van Oers et al., 2015), here we provide one of the first studies investigating diet composition effects on correlated personality traits phenotypic variance (but see Han & Dingemanse, 2017), taken as an important environmental factor explaining part of the non‐genetic differences in behavioural development (Dochtermann et al., 2014). Our results showed that food provisioning affects personality traits in the early life. On one hand, the relative handling stress response, measured as the increase in breath rate over a handling period, was less strong when the number of spiders in the diet was higher. The high nutritional value of spiders has been related to their higher taurine content (55%–60%; Ramsay & Houston, 2003) compared with other woodland arthropods, an amino acid that has been proposed to be one of the most essential substances in the body in birds and mammals (Ripps & Shen, 2012). Taurine is involved in essential processes of neonatal development, as feather growth (Ramsay & Houston, 2003), bone formation (Martin & Patrick, 1961) or the visual system (Neuringer et al., 1990; Palackal et al., 1986), and also stimulates cognitive performance (Arnold et al., 2007). Importantly, taurine can act as a neuroinhibitor of the hypothalamic–pituitary–adrenal (HPA) axis, causing a lower stress response (Engelmann et al., 2003). The HPA axis regulates, in addition to other anatomical structures, adaptive responses to stress in animals via its downstream effectors, the glucocorticoids (Smith & Vale, 2006 and references therein). Thus, a higher content of taurine in the diet of great tit nestlings provisioned with higher number of spiders might explain the reduction of breath rate we found, and play an important role in the development of neuroendocrine stress response and related behavioural traits.

Variation in docility and handling aggression traits were explained by the type of caterpillar that was delivered to the nest. As described by Brommer and Kluen (2012), easily stressed individuals ‘freeze,’ thereby becoming more docile and less aggressive in their response to being handled compared to less easily stressed genotypes. Accordingly, our findings suggest that nestlings provisioned with higher number of noctuids showed an easily stressed phenotype in contrast to those provisioned with higher number of tortricids, which showed less easily stressed phenotype. Therefore, as great tits prefer noctuids in comparison with tortricids (García‐Navas et al., 2013), and based on our hypotheses that nestlings provisioned with a higher quality diet regime will show less stress (lower docility and more aggressive) than those with lower quality food (higher docility and less aggressive), we would have expected opposite results. However, in a natural context in which we can expect great tit parents to flexibly adapt their provisioning strategies to cope with environmental changes (Arvidsson & Matthysen, 2016), it might have been advantageous to feed the nestlings with higher quantity of tortricids compared to noctuids. Despite the latter being bigger, which implies higher quantity of nutrients per item, if the clumped distribution and sedentary behaviour of tortricids and their conspicuousness (i.e. feeding within a shelter constructed by rolling one or more leaves in a tubular fashion; García‐Navas & Sanz, 2011) favoured both their availability and trappability in a critical moment of the behavioural development of nestlings, this might explain the less easily stressed phenotype linked to tortricids number in the diet found here. Also, these results might lead to the idea that under hypothetical suboptimal environmental conditions or an ‘unexpected’ change, those parents that flexibly adapted their foraging preferences to the novel conditions (a non‐preferred but more abundant food source), favoured the development of a less‐stressed behavioural phenotype on their offspring via diet selection. It is also important to stress that we do not imply a causal relationship between the type of caterpillar and the stress response, hence further research is needed to understand what causes these findings.

## 5 CONCLUSIONS

One of the key environmental factors affecting behavioural development during early life is nutrition (Tremmel & Müller, 2013). Here, our work on a population of wild great tits highlights that the variation in provisioning quantity and quality of specific prey types might determine differences on behavioural traits expression in early life, and that the relationship between those behavioural traits occur mainly at the within‐individual level. The stress‐related behavioural traits measured were repeatable as expected, but they did not form an integrated behavioural syndrome including the three traits. Still, we found weak phenotypic correlations (syndromes) between pairs of traits, mainly at the within‐individual level. This indicates that, these behavioural syndromes are mainly driven by the environment (e.g. Dingemanse et al., 2007). The presence of specific important nutrients in the diet, due to their broad functions in development and neuroendocrine stress response (i.e., taurine), as well as the relative contribution of different types of caterpillars (i.e., noctuids, tortricids) that differ in detectability, influenced the development of behaviour. Hence, parental provisioning behaviour, which may be driven by adaptive choices or feeding constraints, have implications for the development of behavioural phenotypes in the offspring. The increased provisioning of different prey types that associated to variation in specific behavioural traits, adds evidence to the idea, that the environment may directly affect the within‐individual behavioural stability, but such effects are often trait‐specific (Han & Dingemanse, 2017). Therefore, we suggest further investigations are needed to confirm whether the results found here are generalisable.

## AUTHOR CONTRIBUTIONS

**Eva Serrano Davies:** Conceptualization (lead); data curation (lead); formal analysis (lead); funding acquisition (equal); methodology (lead); visualization (lead); writing – original draft (lead). **Alba Miguel:** Data curation (supporting); funding acquisition (equal). **Bernice Sepers:** Investigation (equal); writing – review and editing (equal). **Kees van Oers:** Conceptualization (equal); funding acquisition (equal); writing – review and editing (equal).

## FUNDING INFORMATION

E.S.D. and B.S. were supported by open competition grants OCENW.KLEIN.253 and ALWOP.314 to K.O. respectively, funded by The Netherlands Organization for Scientific Research (NWO). AM was supported by the ‘Viveuropa Mobility Programme’ from FGULEM foundation (Spain).

## CONFLICT OF INTEREST STATEMENT

The authors declare that there are no conflicts of interest.

### OPEN RESEARCH BADGES

This article has earned an Open Data badge for making publicly available the digitally‐shareable data necessary to reproduce the reported results. The data is available at https://zenodo.org/records/10495954.

## Supporting information


Data S1.

## Data Availability

All data used in this study are available on zenodo.org, https://zenodo.org/records/10495954 (Serrano‐Davies et al., 2024).
