# Supplementary material for: Early‐life diet composition affects phenotypic variation of correlated animal personality traits
Source: Ecol Evol. 2024 Aug 19;14(8):e11567. doi: 10.1002/ece3.11567 (PMC11333541; doi:10.1002/ece3.11567)
Supplement: Supplementary file 1 — Data S1. [file ECE3-14-e11567-s001.docx]

Supplementary material for: Early-life diet composition determines variation in the development of animal personality

Table S1. Summary of the fixed effects results information for the multivariate model fitted using MCMCglmm: cbind(BR, Docility, HA) ~ trait - 1 + trait: Noctuidae + trait: Tortricidae + trait: Geometridae + trait: Spiders. Posterior means and 95% credible intervals (CI) are shown. Effec. Samp is the effective sample size. Bold indicates estimates whose 95% CI do not encompass zero.

| **traits** | **posterior mean** | **CI** | **Effec. samp** | ***P*** |
| --- | --- | --- | --- | --- |
| BR | 0.007 | -0.147, 0.152 | 4239 | 0.921 |
| Docility | -0.003 | -0.123, 0.116 | 4000 | 0.951 |
| HA | **1.422** | **1.193, 1.641** | **3544** | **< 0.001** |
| BR: Noctuidae | 0.055 | -0.132, 0.227 | 4000 | 0.535 |
| Docility: Noctuidae | **0.158** | **0.0174, 0.295** | **4000** | **0.025** |
| HA: Noctuidae | **-0.255** | **-0.463, -0.042** | **4000** | **0.015** |
| BR: Tortricidae | 0.026 | -0.148, 0.199 | 4000 | 0.754 |
| Docility: Tortricidae | **-0.195** | **-0.336, -0.065** | **4000** | **0.007** |
| HA:Tortricidae | **0.200** | **-0.001, 0.413** | **4000** | **0.047** |
| BR: Geometridae | 0.042 | -0.124, 0.197 | 3770 | 0.592 |
| Docility: Geometridae | -0.012 | -0.130, 0.123 | 4000 | 0.837 |
| HA: Geometridae | 0.056 | -0.138, 0.236 | 4232 | 0.562 |
| BR: Spiders | **-0.257** | **-0.422, -0.094** | **4000** | **0.003** |
| Docility: Spiders | 0.085 | -0.041, 0.218 | 3610 | 0.190 |
| HA: Spiders | -0.064 | -0.255, 0.122 | 4000 | 0.500 |
